# Using a Web-Based Platform as an Alternative for Conducting International, Multidisciplinary Medical Conferences During the Novel COVID-19 Pandemic: Analysis of a Conference

**DOI:** 10.2196/23980

**Published:** 2021-06-09

**Authors:** Po-Jen Ko, Sheng-Yueh Yu, John Chien-Hwa Chang, Ming-Ju Hsieh, Sung-Yu Chu, Jimmy Wei-Hwa Tan, Wan-Ling Cheng, Pei Ho

**Affiliations:** 1 Division of Thoracic & Cardiovascular Surgery Chang Gung Memorial Hospital Linkou Main Branch and Chang Gung University Taoyuan Taiwan; 2 College of Medicine School of Medicine Chang Gung University Linkou Taiwan; 3 Division of Cardiovascular Surgery Dalin Tzu Chi Hospital Buddhist Tzu Chi Medical Foundation Dalin Taiwan; 4 College of Medicine School of Medicine Tzu Chi University Hualien Taiwan; 5 Department of Surgery Chang Gung Memorial Hospital Linkou Main Branch and Chang Gung University Taoyuan Taiwan; 6 Department of Medical Imaging and Intervention Chang Gung Memorial Hospital Linkou Main Branch and Chang Gung University Taoyuan Taiwan; 7 Department of Cardiovascular Surgery Tainan Municipal An-Nan Hospital China Medical University Tainan Taiwan; 8 Department of Nursing Chang Gung Memorial Hospital Linkou Main Branch and Chang Gung University Taoyuan Taiwan; 9 Department of Cardiac, Thoracic and Vascular Surgery National University Health System Singapore Singapore

**Keywords:** web-based conference, live broadcast, medical education, dialysis access, coronavirus, COVID-19, conference, social media, web-based platform, internet, interaction, teleconference

## Abstract

**Background:**

The COVID-19 pandemic has stunted medical education activities, resulting in most conferences being cancelled or postponed. To continue professional education during this crisis, web-based conferences can be conducted via livestream and an audience interaction platform as an alternative.

**Objective:**

The unprecedented COVID-19 pandemic has affected human connections worldwide. Conventional conferences have been replaced by web-based conferences. However, web-based conferencing has its challenges and limitations. This paper reports the logistics and preparations required for converting an international, on-site, multidisciplinary conference into a completely web-based conference within 3 weeks during the pandemic.

**Methods:**

The program was revised, and a teleconference system, live recording system, director system setup, and broadcasting platform were arranged to conduct the web-based conference.

**Results:**

We used YouTube (Alphabet Inc) and WeChat (Tencent Holdings Limited) for the web-based conference. Of the 24 hours of the conventional conference, 21.5 hours (90%) were retained in the web-based conference via live broadcasting. The conference was attended by 71% (37/52) of the original international faculties and 71% (27/38) of the overall faculties. In total, 61 out of 66 presentations (92%) were delivered. A special session—“Dialysis access management under the impact of viral epidemics”—was added to replace precongress workshops and competitions. The conference received 1810, 1452, and 1008 visits on YouTube and 6777, 4623, and 3100 visits on WeChat on conference days 1, 2, and 3, respectively.

**Conclusions:**

Switching from a conventional on-site conference to a completely web-based format within a short period is a feasible method for maintaining professional education in a socially responsible manner during a pandemic.

## Introduction

The COVID-19 outbreak was first reported in Wuhan, China, in December 2019, and it rapidly developed into a pandemic within 3 months [1]. It poses a significant threat to global health. Travel restrictions, bans on mass gatherings, and social distancing are some of the main control measures that have been adopted in many countries. As a consequence, almost all medical conferences have been cancelled or postponed since February 2020.

Dialysis Access Synergy (DASy)—the official academic conference of the Society of Dialysis Access Specialists (SoDAS) that was organized in conjunction with the Taiwan Association of Vascular and Access Health and Chang Gung University—is a multidisciplinary international meeting that focuses on dialysis access. The conference was scheduled to be held from March 13 to March 15, 2020, in Taoyuan, Taiwan. By mid-February 2020, 90 international and local faculties had committed to participating in the on-site meeting. More than 300 delegates had registered for the conference. However, in late January 2020, the Taiwan government imposed stepwise strategies to contain the viral outbreak, including the Entry Quarantine System, which was implemented on February 14 and targeted many Asian countries [2]. These travel restrictions prevented participants from pandemic-affected countries from attending the conference. Furthermore, those arriving from regions that were not covered by the Entry Quarantine System had an elevated risk of contracting COVID-19 during travel or conference gatherings. The goal of advancing professional education conflicted with the goal of curbing the pandemic.

Due to the rapidly evolving global situation, the DASy organizing committee and SoDAS executive committee had to choose between abandoning the conference after a year-long preparation period or continuing with the planned conference and bearing the substantial health risk. Eventually, the committees chose to continue with the conference as scheduled but converted it into a completely web-based format by using teleconference technology and livestreaming the conference over social media. The entire switch was accomplished within 3 weeks. In this paper, we share our experience with this process.

## Methods

**Figure 1:**
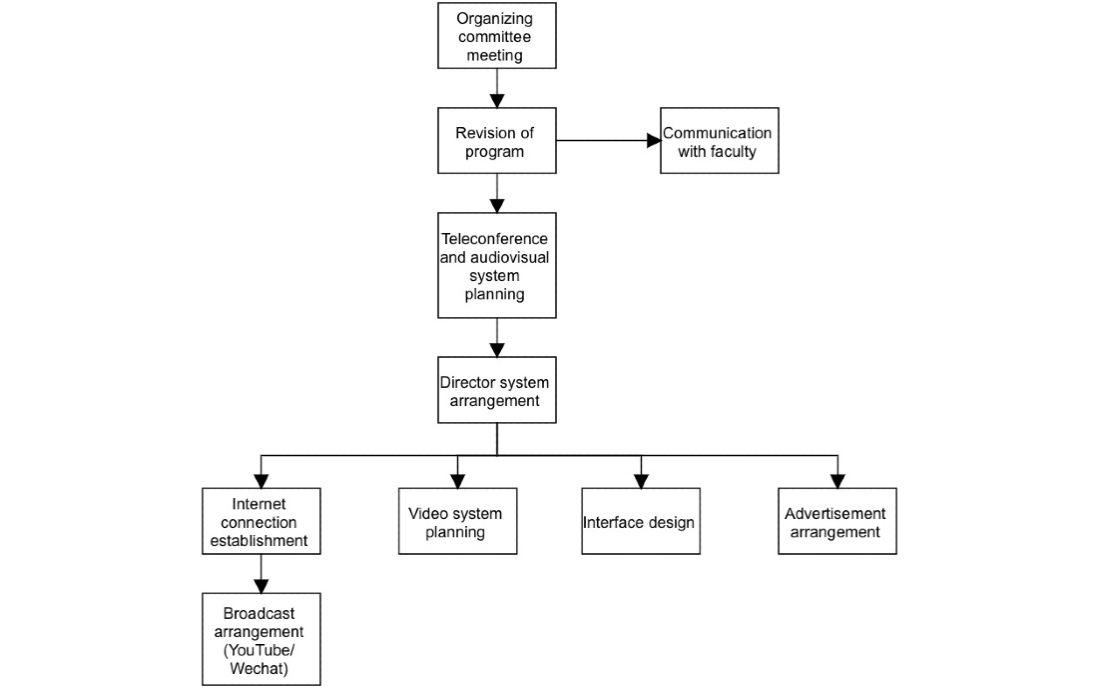
Flowchart of the logistical issues involved in converting an on-site meeting into a web-based conference.

Several logistical and practical challenges must be resolved to convert an on-site, in-person conference to a completely web-based format. Such challenges include program revision; venue adjustment; teleconference, audiovisual, recording, and live broadcasting arrangements (Figure 1); and the promotion of the conference and audience engagement.

### Program Revision

During the pandemic, many faculty members who were clinicians were busy with the management of the sudden health crisis in their home countries and were unable to prepare for the conference. In addition, they may have been unable to join the real-time teleconference discussion because of time zone differences or work schedule conflicts. Facilitating efficient communication for engaging the faculty members and for confirming their degree of commitment was the first task of the conversion to a web-based conference.

Teleconferencing has its limitations. Hands-on skills teaching typically cannot be effectively conducted via a web-based platform. Dialysis access involves many procedural and interventional skills. The DASy committee removed the precongress workshops, which was the main session for skills training. Conversely, after assessing the immanent need of medical knowledge during the unprecedented COVID-19 health crisis, the committee added a special session—“Dialysis access management under the impact of viral epidemics.” In this ad hoc session, dialysis access clinicians who were working in the epicenter of Wuhan and other Chinese cities as well as representatives of other countries presented measures that were taken to maintain dialysis access and protect health care worker safety. Furthermore, national policy, institutional workflow, protocols, and patient flow control were discussed.

### Teleconference Platform

The teleconference required a real-time stable system, audiovisual clarity, a user-friendly platform, the accommodation of numerous participants, and broadcasting ease. The organizing team reviewed several available options and decided to use the Zoom cloud meeting system (Zoom Video Communications Inc). Conference speakers and session panelists were required to download the Zoom meeting app on their computers or mobile phones. A total of 2 Zoom cloud meeting rooms were established—1 for the actual conference and 1 for conference preparation. The preparation room was used to ensure that speakers were connected. The actual conference room was used during talks and discussions and was broadcasted live. A rehearsal, which involved the organizing host, audiovisual team, broadcast director, and 14 available faculty members, was conducted 1 week before the actual meeting.

### Venue

A web-based conference requires a much smaller venue than a physical conference. Hence, a large auditorium was not required, and a 90-m^2^ room in the Fullon Hotel Linkou at Taoyuan city was used instead. This new venue housed the 10 to 15 organizers, local faculty members, and the audiovisual team and their equipment. The venue setup included a presentation podium, a table for chairpersons, and a table for moderators (who were physically present in Taiwan). Adequate social distancing was required inside the meeting room. The previously planned industry sponsor exhibition hall and slide preview room were omitted. Short videos that introduced sponsors’ devices were shown between the conference sessions. Another small room was required to accommodate the broadcast director, his console, and the conference equipment.

### Teleconference Proceedings

**Figure 2:**
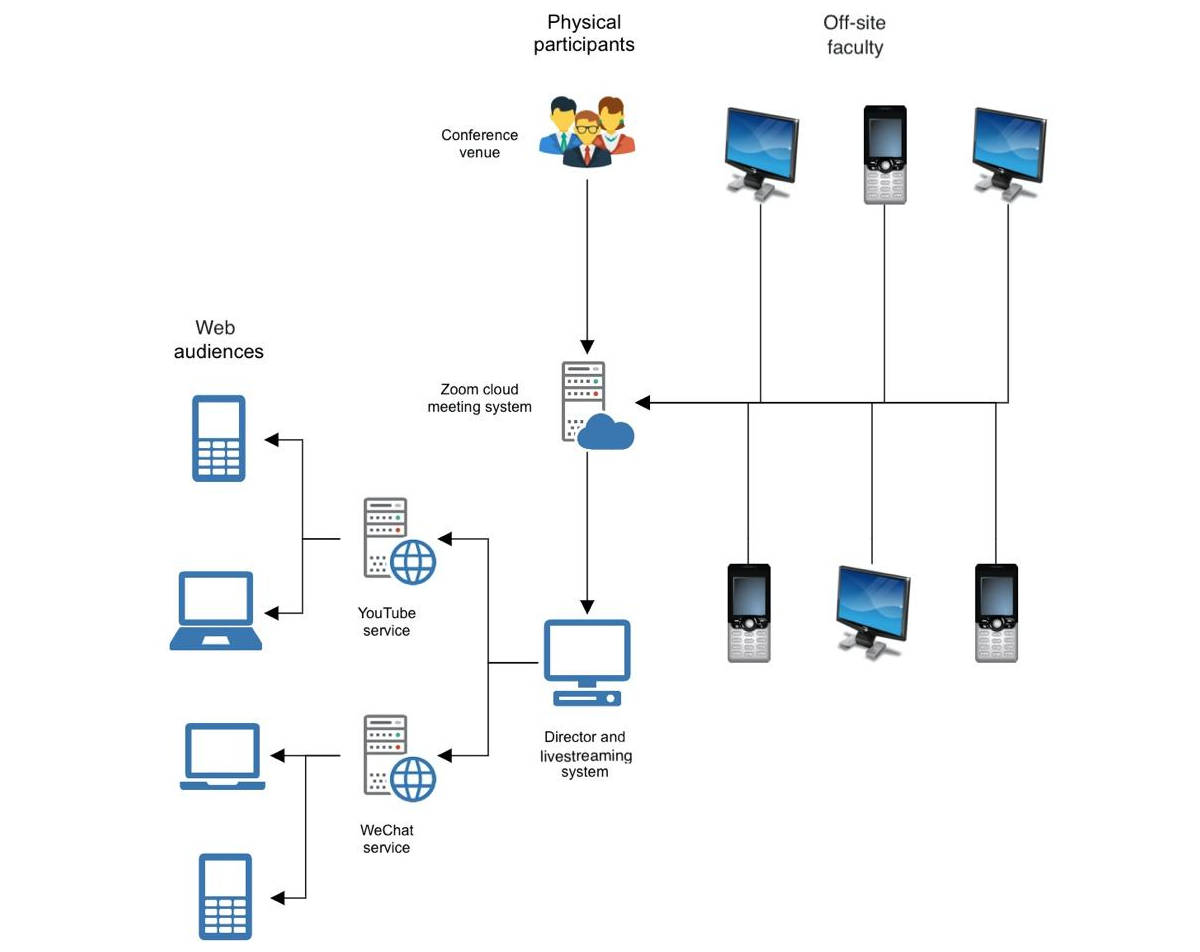
A schematic of the process of broadcasting a live, web-based conference by using teleconferencing technology.

The broadcast director was responsible for conference time keeping and audiovisual signal assignment. The camera was positioned to capture the presentation podium, chairpersons, or moderators during the discussion sessions. All faculties were requested to provide a prerecorded presentation to avoid any complications when files were switched during the conference. Few faculties provided their presentations on the meeting date and thus made use of the screensharing function to present live talks. All nonphysically present speakers and moderators joined the meeting through Zoom. The main screen broadcasted the conference agenda and showed the PowerPoint video, which included prerecorded narration; real-time faculty presentations; the master screen of all faculties who had logged onto Zoom; or Zoom images of selected faculty members during question-and-answer sessions. In the preparation room, the assistant of the broadcast director liaised with faculty members to prepare them for joining the meeting at the appropriate time. Afterward, the conference continued in accordance with the program agenda and was livestreamed to the web audience (Figure 2).

### Live Broadcasting

To reach a worldwide audience and achieve educational goals, the DASy and SoDAS committees decided to provide a free live broadcast over two easily accessible social media platforms—YouTube (Alphabet Inc) and WeChat (Tencent Holdings Limited). Links to the livestream of DASy 2020 were generated and disseminated 10 days before the conference.

### Web-Based Conference Promotion and Audience Engagement

Teleconferencing can be promoted through email communication, website announcements, and social media chat groups. Due to time constraints, promotion opportunities for the web-based DASy conference were limited. Nonetheless, the Japan Endovascular Treatment website announced the web-based DASy 2020 conference to its members as a gesture of support.

During the live broadcast, both the YouTube and WeChat platforms provided a real-time, web-based, text-response mechanism. A selected member among the chairpersons was responsible for collecting the questions and comments from the web audience and discussing them during the session. This was done to maximize the engagement of the web audience during the event.

Both the YouTube and WeChat platforms allowed individuals to view the conference regardless of whether they had a registered account. On the YouTube platform, the number of visits and gross geographic locations were recorded, and feedback was provided to the organizers. On the WeChat platform, in addition to the number of visits and geographic locations, the service provider that was responsible for setting up the livestream captured users’ first and last login times and their cumulative viewing duration.

## Results

The DASy 2020 conference was held in Taiwan as scheduled and broadcasted worldwide. The total duration of this web-based conference was 2.5 days. The conference had a total livestreaming time of 21 hours and 33 minutes (the original on-site conference time was 24 hours). A 2-hour session titled “Dialysis access management under the impact of viral epidemics” involved 6 speakers from 4 countries, including Wuhan, China, and resulted in stimulating discussions on this topic. The precongress workshops and grand challenge competitions were cancelled.

In total, 52 international faculties and 38 local Taiwanese faculties confirmed that they were going to attend the original physical meeting. The web-based conference was attended by 38 overseas faculty members (37 web attendees and 1 physically present attendee) and 27 local faculties (13 web attendees and 14 physically present attendees). The international faculties that participated were from Australia, Canada, China, Germany, Greece, the Hong Kong Special Administrative Region, Japan, Malaysia, Singapore, South Korea, Thailand, and the United States. The only international faculty that was physically present was from Indonesia; at the time of the conference, the impact of the pandemic was less serious in South East Asia. Switching the conference to a web-based format resulted in the successful, active participation of 71% (37/52) of our international faculties and 71% (27/38) of the overall faculties in the conference during the COVID-19 crisis. Faculty members were from multiple disciplines and included vascular surgeons, nephrologists, intervention radiologists, urologists, dialysis nurses, and engineers. Originally, 66 presentations were planned for the main conference. However, with the web-based format, 61 out of 66 planned presentations (92%) were delivered. None of the main conference sessions were cancelled.

On the first day of the event, the conference received 1810 and 6777 visits on YouTube and WeChat, respectively. On the second and third days, the conference received 1452 and 1008 visits on YouTube, respectively, and 4623 and 3100 visits on WeChat, respectively. The total number of visits to the DASy 2020 live broadcast was 13,302. The visits continued to increase during the conference livestream. Individuals from Asia, the Middle East, Australia, Europe, and North America attended the web-based conference.

On the WeChat platform, 1605 individuals with a registered account viewed the live broadcast. Of these individuals, 312 watched the broadcast for >10 minutes. The numbers of identified users who had watched the livestream for 10 minutes to 1 hour, 1 to 3 hours, 3 to 10 hours, and >10 hours were 174, 69, 49, and 20, respectively. On the basis of the first and last login dates of the identified users, 26% (82/312) attended all 3 days of the conference, 27% (84/312) attended 2 of the 3 conference days, and 47% (146/312) only attended 1 conference day.

## Discussion

### Principal Findings

Globalization, crossover, and multidisciplinary collaboration are effective strategies for advancing health care services in various fields. These require human interaction—preferably, face-to-face interactions—in varying group sizes. With the ease of travel and simplification of short-term entry requirements in many countries, international medical conferences represent an essential modality of professional education as well as incubators for new ideas about service improvement and scientific research. DASy, which is a multidiscipline, multinational, dialysis access–focused meeting, has embraced this concept. The DASy program involves auditorium presentations, podium discussions, hands-on skills training, and competitions based on specific themes. The preparatory work of DASy 2020 started in April 2019. However, the meeting time coincided with the COVID-19 outbreak. This potentially lethal infectious disease posed huge challenges to providing health care education through conventional meetings. Since February 2020, most international medical conferences have been either cancelled or postponed [3,4]. Indeed, the spread of COVID-19 has been reported to be attributable to business meetings [5]. The health care community could opt to forgo training and education during this pandemic or endanger participants’ lives with mass gatherings. We considered neither of these approaches desirable. Therefore, we attempted to convert a physical conference into a completely web-based format and aimed to continue the effort of promoting medical education without imposing additional risks to the participants. Moreover, this approach fulfilled our social responsibility of restricting disease transmission.

The COVID-19 outbreak resulted from a novel strain of coronavirus with poorly characterized virulence, transmission modes, and infectivity. It first affected the city of Wuhan. Afterward, it spread throughout China before affecting nearly the whole world. The outbreak statistics, travel warnings, travel bans, and compulsory quarantine requirements in various countries have changed rapidly. With such an unprecedented infectious disease crisis, time was a major challenge for conference organizers. However, the time to act played a vital role in managing such a challenge. For DASy 2020, although the organizing committee discussed the option of holding the meeting on the internet, the decision to do so was made only 3 weeks before the meeting. To achieve this task within 3 weeks, the organizing team required strong support from the faculty members, the adoption of readily available telecommunication and live broadcast technology, information technology and audiovisual experts, and efficient promotion.

Teleconferencing technology has been increasingly used in patient care and medical education and has been proven to be beneficial [6,7]. Good program content and quality audiovisual platforms are essential elements of web-based conferences [8]. To minimize the number of potential technical and reception interruptions, all participating faculties were asked to upload a prerecorded PowerPoint file with narration in advance. The few speakers who could not do so delivered their talks via livestreaming through the Zoom system and shared their screen. The use of the preparation room helped to address any complications during the actual conference.

Many social media and platforms support livestreaming and instant chat functions, including YouTube, Facebook, Instagram, Twitter, Vimeo, and Podcasts. YouTube was selected because it is among the top 2 platforms in terms of user penetration in Western countries [9]. Although YouTube is readily accessible and is a popular platform for viewing video content in many parts of the world, it is inaccessible in some Asian countries such as China, which has seen a rapid increase in the need for dialysis over the past decade [10]. Hence, WeChat was selected as an additional broadcast platform. WeChat is a multifunctional social media app that is user friendly and popular in China. Other options included Tencent online video, Youku, Taobao live, Sina Weibo with Tencent online video, and Taobao live with additional payment functions. By broadcasting simultaneously over these the YouTube and WeChat platforms, organizers maximized the accessibility of the conference for audiences worldwide. Therefore, during the 2.5 days of the conference, a total of 13,302 visits were recorded, which was considerably higher than the number of previous DASy conference attendees (350-400 delegates). Thus, more interested individuals may be reached through web-based conferences than with similar on-site conferences. However, free registration, the convivence of web-based viewing, and distractions from individuals’ environments could result in a wide range of attention to conference presentations. On the basis of the WeChat data, numerous individuals viewed the conference for <10 minutes. Although these individuals contributed to visit counts, they were unlikely to have seriously attended the conference. Nonetheless, the number of web participants who viewed the conference for >10 minutes (n=312) was still approximately 15 times the number of on-site conference delegates in 2019, which was approximately 20.

Since the occurrence of the COVID-19 pandemic in early 2020, the landscape of medical conferences has considerably changed (ie, from being postponed or cancelled to being conducted via web-based methods). The methods and technology used for conducting web-based meetings have consequently evolved and diversified within a short period. Conference organizers may choose the format and platform that best suits the following three objectives: (1) providing knowledge and information on what they aim to convey, such as didactic lectures, focused expert discussions, live procedures, or competitions; (2) reaching their target audience size and geographical location; and (3) obtaining funding and resources. The platforms that are being used for web-based conferences include comprehensive commercial solutions, mixed commercial solutions, and custom-made platforms. Comprehensive commercial solutions (eg, EventMobi and Remo Conference) can handle many aspects of web-based conferences, including registration, web-based conference spaces, event app building, instant polling and question-and-answer sessions, in-app chats, interactions with sponsors, and data analytics. With regard to mixed commercial solutions (eg, Zoom conferences and WeChat livestreaming), conference organizers could select a specific platform for web-based conferences, livestreams, registration, and other functions based on the requirement of the individual event. For recurring web-based conferences or workshops with specific audiovisual or interaction requirements, a custom-made platform may be preferred. The time and cost required for building these three types of web-based platforms also vary. Depending on the situation of the pandemic, some medical conferences may also consider using a hybrid solution (ie, integrating on-site and web-based meetings). Facilitating interactions between on-site and off-site faculties and participants requires careful planning.

Although web-based conferences could attract numerous audience members across a wide geographic area, limitations remain. First, skills training is an important objective of medical conferences; it is usually conducted in small group workshops and is seldom successfully delivered through a web-based modality. Second, vigorous interactive discussions among the faculties and delegates, which are common in conferences, are difficult to replicate in web-based platforms. Third, instant polling from the delegates (web audience) for opinion and practice surveys was impossible to conduct over the platforms used for DASy 2020. Obtaining conference evaluations and feedback is difficult. Fourth, delegates could not evaluate new devices and technology that were relevant to their practices and engage with industry representatives during the web-based conference. Furthermore, whether participants were exempted from work hours at their workplace for the web-based conference was uncertain.

Some of these limitations might be overcome with advances in technology for web-based meetings. For example, web-based controlled simulator training might be used for skill development, electronic polling may be conducted through a mobile app or specifically designed platform, and industry booths could incorporate video demonstrations. However, specific limitations with regard to web-based conferencing are likely to arise in the foreseeable future. Therefore, web-based conferences cannot efficiently replace on-site meetings but could be a feasible and reliable alternative during unpredictable times, such as the COVID-19 pandemic. Indeed, given the aforementioned advantages, web-based conferences could be incorporated into all on-site conferences wherever possible. Attention should be paid by conference organizers to web-based conference delegates’ experiences, engagement, interactions, and feedback, so as to maximize the benefits of a web-based platform. Moreover, on-site conferences may incorporate some web-based presentations and discussions by prominent faculties that have difficulty with physically attending the meeting. This would enable the conference organizers to enrich the educational content of the meeting in a highly versatile manner.

The organizing committee understood the limitations of web-based conferencing. Hands-on courses and the grand challenge competition were cancelled to make room for topics that were the most relevant to worldwide dialysis health care workers facing the imminent threat of COVID-19. The “Dialysis access management under the impact of viral epidemics” session on the first day of the event involved the first-hand experiences of faculty members from various areas that were hit hard by COVID-19, particularly Wuhan. Meeting analytics revealed that a much higher number of visits to the web-based conference occurred on day 1 than on days 2 and 3. This reflected the world’s interest in the topics that were covered during the day-1 session.

Time zone differences represented another challenge for the speakers at DASy 2020. Adjusting the program sequence may have reduced the extent of the problem by some extent. At DASy 2020, several speakers remained on Zoom during out-of-work hours; 1 faculty member stayed up until 3 AM (local time). Several speakers opted to submit audio-recorded presentations only because they were unable to join the live sessions.

The internet was derived from technology that was invented by the US government to cope with the threat of the Soviet Union during the Cold War era [11]. During the COVID-19 pandemic, pathogens are the major problem. Internet technologies are extremely effective tools for tackling some of the major challenges posed by pandemics. Maintaining the ability to disseminate high-quality medical education can be achieved through web-based conferencing, even during an unexpected, sudden, global health crisis.

Due to the time and budget constraints of the conversion to a web-based conference, web-based polling and audience feedback mechanisms were not implemented. The conference live broadcast was open to all people and did not require preregistration. Thus, a postconference follow-up was impossible. Furthermore, the demographic and viewing data of audience members were incomplete. We suggest that a feedback mechanism and polling system should be considered for every web-based event.

### Conclusion

On the basis of our experience with DASy 2020, switching a conference from on-site operations to web-based operations within a relatively short period while maintaining its quality is possible. A web-based medical conference that was conducted during the pandemic delivered the educational goal without risking the safety of individuals. We recommend that organizing committees of future medical conferences should consider switching to a web-based format in the event of unexpected epidemics of infectious diseases. Furthermore, we recommend that all medical conferences broadcast at least a portion of their meeting content via the internet to broaden their educational value worldwide.

## Abbreviations

DASy: Dialysis Access Synergy
SoDAS: Society of Dialysis Access Specialists
